# Family medicine residents’ attitudes toward mental illness

**DOI:** 10.1192/j.eurpsy.2023.1895

**Published:** 2023-07-19

**Authors:** A. Jaoua, M. H. salhi, A. ben haouala, L. gaha

**Affiliations:** 1 monastir tunisia; 2 Monastir Tunisie, Hôpital Fattouma Bourguiba Monastir; 3 monastir tunisia, fattouma bourguiba hospital monastir, mourouj 1, Tunisia

## Abstract

**Introduction:**

The stigma of mental illness is a complex socialphenomenon that is widespread throughout the world, even amonghealth care professionals.

**Objectives:**

Assessing attitudes towards mental illness among familymedicine residents in Tunisia

**Methods:**

This is a descriptive cross-sectional study among of familymedicine residents enrolled at the Faculty of Medicine in Monastir(Tunisia), conducted over a period of 3 months (July 2022 to October2022). The CAMI (Community Attitudes towards the Mentally Ill)scale was used to assess the attitude towards mental illness. Sociodemographic data were collected through a pre-established questionnaire. The data were analyzed using SPSS software 26 thversion.

**Results:**

Our population was made up of 95 family medicineresidents, divided into 28 males and 67 females. Sex ratio was 2.39. The average of age was 28 years with extremes 25 and 35 years.46.3% (n=44) of the residents were enrolled in the first year, 22.1%(n=21) enrolled in the second year and 31.6% (n=30) enrolled in thethird year. 88.4% (n=84) of the residents did a psychiatric rotationduring their training.We found that 47.4% of residents (n=45) had a positive attitudetowards mental illness.

**Conclusions:**

Improving the attitudes of primary care physicianstowards people with mental illness is necessary to provide goodquality care to these patients

**Disclosure of Interest:**

None Declared

